# Healthcare trajectory of children with rare bone disease attending pediatric emergency departments

**DOI:** 10.1186/s13023-019-1284-1

**Published:** 2020-01-03

**Authors:** David Dawei Yang, Geneviève Baujat, Antoine Neuraz, Nicolas Garcelon, Claude Messiaen, Arnaud Sandrin, Gérard Cheron, Anita Burgun, Zagorka Pejin, Valérie Cormier-Daire, François Angoulvant

**Affiliations:** 1 Assistance Publique - Hôpitaux de Paris, Pediatric Emergency Department, Necker-Enfants Malades Hospital, Paris Descartes University - Sorbonne Paris Cité, Paris, France; 2 Assistance Publique - Hôpitaux de Paris, Departement of Genetics, National Reference Center for Skeletal Dysplasia Hôpital Necker-Enfants Malades, Paris, France; 3 Département de Génétique, Université Paris Descartes-Sorbonne Paris Cité, INSERM UMR1163, Institut IMAGINE, Hôpital Necker-Enfants Malades, Paris, France; 4 INSERM, Centre de Recherche des Cordeliers, UMRS 1138, Université Paris Descartes, Sorbonne Paris Cité, Paris, France; 5 Assistance Publique - Hôpitaux de Paris, Department of Medical Informatics, Necker-Enfants Malades Hospital, Paris Descartes University, Sorbonne Paris Cité, 75015 Paris, France; 6 Institut IMAGINE, Plateforme de Data Science, Université Paris Descartes, Sorbonne Paris Cité, Paris, France; 7 Banque Nationale de Données Maladies Rares, Hôpitaux de Paris, Hôpital Necker-Enfants Malades, Paris, France; 8 Hôpitaux de Paris, Department of Pediatric Orthopedics, Necker-Enfants Malades Hospital, Paris Descartes University, Sorbonne Paris Cité, 75015 Paris, France

**Keywords:** Rare disease/pathology, Bone disease/pathology, Healthcare delivery, Pediatric emergency medicine, Multiple chronic medical conditions

## Abstract

**Background:**

Children with rare bone diseases (RBDs), whether medically complex or not, raise multiple issues in emergency situations. The healthcare burden of children with RBD in emergency structures remains unknown. The objective of this study was to describe the place of the pediatric emergency department (PED) in the healthcare of children with RBD.

**Methods:**

We performed a retrospective single-center cohort study at a French university hospital. We included all children under the age of 18 years with RBD who visited the PED in 2017. By cross-checking data from the hospital clinical data warehouse, we were able to trace the healthcare trajectories of the patients. The main outcome of interest was the incidence (IR) of a second healthcare visit (HCV) within 30 days of the index visit to the PED. The secondary outcomes were the IR of planned and unplanned second HCVs and the proportion of patients classified as having chronic medically complex (CMC) disease at the PED visit.

**Results:**

The 141 visits to the PED were followed by 84 s HCVs, giving an IR of 0.60 [95% CI: 0.48–0.74]. These second HCVs were planned in 60 cases (IR = 0.43 [95% CI: 0.33–0.55]) and unplanned in 24 (IR = 0.17 [95% CI: 0.11–0.25]). Patients with CMC diseases accounted for 59 index visits (42%) and 43 s HCVs (51%). Multivariate analysis including CMC status as an independent variable, with adjustment for age, yielded an incidence rate ratio (IRR) of second HCVs of 1.51 [95% CI: 0.98–2.32]. The IRR of planned second HCVs was 1.20 [95% CI: 0.76–1.90] and that of unplanned second HCVs was 2.81 [95% CI: 1.20–6.58].

**Conclusion:**

An index PED visit is often associated with further HCVs in patients with RBD. The IRR of unplanned second HCVs was high, highlighting the major burden of HCVs for patients with chronic and severe disease.

## Background

In Europe, a disease is considered rare if it affects fewer than 1 person per 2000. There are more than 6000 rare diseases and the number of known rare diseases is continually increasing. Rare diseases affect 30 million European citizens [1]; 80% of these diseases have a genetic etiology and 75% are pediatric diseases [2]. The management of rare diseases raises multiple issues concerning diagnosis, follow-up, treatment, acute incidents, complications, dependence, impact on families, and the skill of healthcare professionals [3]. Many children with rare genetic diseases fulfill the criteria for medical complexity, resulting in specific healthcare needs, such as multidisciplinary hospital-based comprehensive care programs [4].

Many studies have shown that children with genetic diseases and those with medically complex diseases account for a significant proportion of pediatric hospital admissions [3, 5–10]. Mortality is higher in this population [6, 10–12], whose hospital stays are longer and more expensive [3, 6, 10, 11]. Nevertheless, most published studies have focused on hospitalized children, with very few reports relating to genetic disorders in primary care, particularly in emergency structures. The precise role of the PED in the healthcare trajectory of patients with rare diseases remains unknown, and a better understanding of this role is required to improve patient care and management. Rare bone diseases (RBDs) particularly complex and characterized by an intensive use of primary care facilities [13]. RBDs constitute a heterogeneous group of rare genetic diseases with skeletal, respiratory, neurological, and visceral impairment, inducing physical, sensory and intellectual disabilities of various degrees of severity [14]. Osteogenesis imperfecta (OI) is the most frequent RBD [13].

New tools have recently been shown to be of value for describing and identifying patients with rare diseases in clinical data warehouses. These tools make it possible to handle large set of data, particularly for rare and chronic complex diseases, and to describe the healthcare trajectory of the patients, an essential step in the improvement of interventions [15–17].

The aim of this study was to analyze the place of the pediatric emergency department (PED) in the healthcare trajectories of children with RBD, in terms of the incidence of healthcare visits (HCVs) within 30 days of an index PED visit. We also analyzed the incidence of planned and unplanned HCVs and the proportion of patients considered to have chronic medically complex (CMC) conditions at their PED visit. We developed a graphical representation of a department-oriented healthcare trajectory model, according to which 25% of children with RBDs visiting the PED are likely to attend another HCV in the following month.

## Materials and methods

### Study setting and design

We set up a retrospective single-center cohort study to describe the healthcare profiles of patients with RBD attending the PED from January 1 to December 31, 2017.

This study was conducted at Necker Enfants-Malades Hospital, a university hospital specializing in the care of children with rare diseases. This hospital has a number of strengths in this field: (i) it hosts the “IMAGINE – Institut des Maladies Génétiques”, an institute for genetic diseases, together with 35 national reference centers for rare diseases, including a reference center for rare bone diseases (RBDs); (ii) it has a clinical data warehouse with powerful tools (Dr Warehouse®) developed by the IMAGINE Institute [1]; (iii) it houses a PED that manages 78,000 patients per year, and previous studies (personal data) have shown that 40% of the patients attending the PED have an underlying chronic condition of genetic origin.

### Participants

The inclusion criteria were: child under the age of 18 years admitted to the PED of NEM Hospital in 2017 with a suspected or confirmed RBD registered by the reference center for RBDs. The exclusion criterion was the disrespect of any one of the inclusion criteria.

### Classification of healthcare and PED visits

**Fig. 1:**
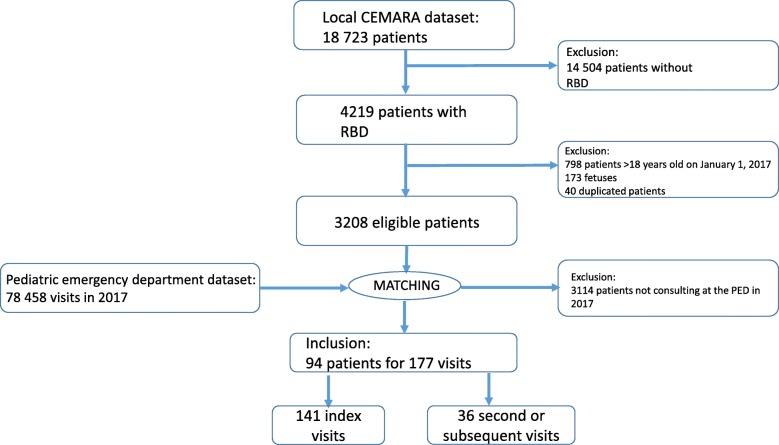
Flow chart for the identification of patients with rare bone diseases

PED visits were considered to be index visits if the patient had not visited a PED in the previous 30 days. Previous HCVs were defined as visits to another doctor or department occurring within the seven days preceding the PED visit. Second HCVs were defined as planned and unplanned visits occurring within 30 days after the index PED visit, and subsequent HCVs were defined as visits occurring within 30 days after the second HCV or a former subsequent HCV (Fig. 1).

### Data collection

#### Step 1: patient identification

We identified patients with suspected or confirmed RBD from CEMARA (CEntre des MAladies RAres), a national online registry database launched in 2007 by the French reference centers for rare diseases network. CEMARA records information on the epidemiological features of rare diseases and related medical activities from reference centers. A minimum dataset (including the diagnosis) was collected for all the patients with registered rare diseases [18, 19]. The CEMARA diagnosis ontology is based on the Inventory of Rare Diseases developed by ORPHANET [18]. At the PED, URQUAL v5® software (McKesson Corp., Paris, France) was used for the prospective recording of data for the patients. We identified all patients with RBDs who consulted at the PED by cross-matching URQUAL® data with the data from the local CEMARA database.

#### Step 2: extraction of clinical data

We extracted clinical information from paper medical records for the patients identified at the PED from January 1 to December 31, 2017. The principal items of information extracted were sociodemographic parameters; number of PED visits since birth; chief complaints; previous HCVs; whether the patient was identified as having a RBD in the medical records of the PED; additional healthcare consumption during the PED visit defined as laboratory tests, medical imaging, nursing care, intervention of the medical subspecialist; visit outcome; planned and unplanned HCVs within 30 days after the index visit.

Data concerning the patient’s medical history, RBD diagnosis, comorbidity, technological dependence, and longitudinal tracking data for the patient’s healthcare trajectory were extracted from the hospital data warehouse with Dr. Warehouse® (DrWH), a document-based, free text-oriented clinical data warehouse software suite. DrWH combines 21 sources of data from the NEM Hospital. It includes a full-text search engine, and, as of June 2018, it contained more than 4.6 million clinical free-text documents for more than 548,000 patients. DrWH also includes a patient-centered view, making it possible to explore the medical history of a single patient with a search engine restricted to the patient’s documents and a searchable timeline. With this tool, it is possible to explore patient history and to trace the patient’s healthcare trajectory before and after their visit to the PED [15, 17]. By cross-matching DrWH data with paper medical records from the PED, we were able to retrace the healthcare trajectories and medical histories of each patient. Two members of the research team reviewed the records of each patient together, to identify cases of medical complexity.

### Classification of the patient’s condition

The main outcome of interest was the incidence of second HCVs within 30 days after the index visit. The secondary outcomes were: incidence of planned and unplanned second HCVs, number of previous HCVs before the index PED visit, healthcare consumption during the PED visit, proportion of patients considered to have chronic medically complex (CMC) conditions, and the impact of CMC status on second HCVs. The underlying conditions were classified into three categories — chronic medically complex; chronic non-medically complex; non-chronic non-medically complex —; according to the classification proposed by Simon et al. [20] (Additional file 1). Children were considered to have a CMC condition if they had (i) significant chronic conditions affecting two body systems; (ii) a progressive condition associated with deteriorating health and a decreased life expectancy in adulthood; (iii) continuous dependence on technology for at least six months; or (iv) progressive or metastatic malignancies affecting life functions. Non-CMC conditions were defined as chronic conditions lasting at least one year, involving a single body system and not progressive. Children non-chronic and non-medically complex conditions were defined as (i) children with acute nonchronic conditions or (ii) healthy children.

### Statistical analysis

Statistical analysis was performed with Stata v13.1© and R v3.3.3®. Categorical data are reported as numbers and percentages, and continuous data are reported as means and SD or medians and interquartile values. Poisson regression analysis was performed to calculate the 95% confidence interval of the incidence of second HCVs. A Poisson regression model was used to estimate the incidence rate ratio (IRR) of second HCVs (dependent variable) between patients with CMC and non-CMC conditions (independent variable), with adjustment for age (independent variable).

### Ethics and security

The study data were anonymized and secured. The information was collected and managed with REDCap® tools [21] on a server within the IMAGINE Institute. Data collection, storage and the secondary use of CEMARA, the URQUAL® Emergency database and DrWH were approved by the French National Commission for Data Protection and Liberties [15, 18]. The study was approved by the institutional review board (CENEM) of Necker Enfants-Malades University Hospital, AP-HP.

## Results

### Identification of RBD patients

**Fig. 2:**
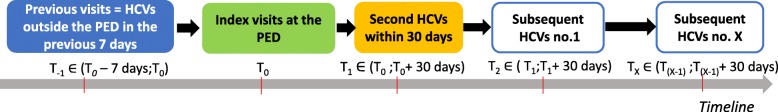
Healthcare trajectory of children with rare bone diseases in the pediatric emergency department

We identified a total of 4219 RDB patients in the local CEMARA dataset. We excluded 798 patients because they were more than 18 years old on January 1, 2017. We also excluded 173 fetuses and 40 duplicated patients. We selected 3208 patients for matching with the PED database, which included data for 78,458 visits from January 1 to December 31, 2017. This cross-matching identified 94 patients who attended 177 visits in total in 2017 (Fig. 2). In 2017, 782 patients with active RDB were managed by the reference center (at least one visit to the hospital per year). Thus, 12% of the patients with active RDB visited the PED in 2017.

### Population characteristics

The median age of the patients was seven years (Q1: 5 years; Q3:13 years), and 61% (*n* = 57) of the patients were male. The median number of visits to the PED of the study hospital since birth was 3 (Q1: 2 visits; Q3: 9 visits), and 97% (*n* = 91) of the patients lived in the Parisian region, the catchment area of the hospital (Table 1).

**Table 1:** Demographic characteristics

Characteristics	Study population (*n* = 94)
Median age (years) [Q1-Q3]	7 [5–13]
Male	57 (61%)
Median number of visits to PED since birth [Q1-Q3]	3 [2–9]
Home address within the hospital’s catchment area	91 (97%)
Patient with osteogenesis imperfecta	37 (39%)
Technological dependence	16 (17%)
Chronic medically complex condition	35 (38%)

The RBD most frequently diagnosed in these patients was osteogenesis imperfecta, which accounted for 39% (*n* = 37) of all patients. The other RBDs identified corresponded to 45 different diseases, 80% of which affected only one patient (Additional file 2). More than half patients (52%; *n* = 49) had at least one comorbid condition (Table 1). Technological dependence (e.g. use of a wheelchair, gastrostomy, noninvasive ventilation or a hearing aid) was noted for 17% (*n* = 16) of the study population. Overall, 38% of the patients (*n* = 35) were considered to have chronic medically complex conditions (Table 1).

### Healthcare trajectory

There were 177 visits in total: 141 were classified as index visits, and the other 36 PED visits were preceded by an index visit in previous 30 days and were therefore classified as second or subsequent HCVs (Fig. 2).

**Fig. 3:**
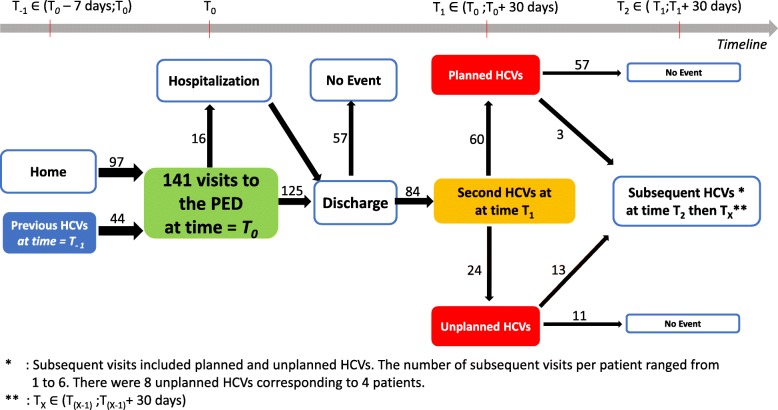
*: Subsequent visits included planned and unplanned HCVs. The number of subsequent visits per patient ranged from 1 to 6. There were 8 unplanned HCVs corresponding to 4 patients. **: T_X_ ***∈*** (T_(X-1)_ ;T_(X-1)_+ 30 days)

The outcome of the index visit to the PED was hospital discharge in 89% of cases (*n* = 125). The rate of hospitalization in short-stay units (SSUs) was 6% (*n* = 8); six of the patients were then discharged and the other two were subsequently admitted to a conventional ward. The rate of hospitalization rate in conventional wards was 7% (*n* = 10), including the two patients transferred from SSUs. The overall rate of hospitalization was 11% (*n* = 16). In total, 44 of the 141 index visits to the PED (31%) were preceded by a HCV to another doctor or department in the previous seven days, and 26 (18%) of the patients were referred by a healthcare professional. After the index visit, there were 84 s HCVs, corresponding to an incidence rate of 0.60 [95% CI: 0.48–0.74]. These second HCVs included 60 planned (incidence = 0.43 [95% CI: 0.33–0.55]) and 24 unplanned (incidence = 0.17 [95% CI: 0.11–0.25]) HCVs. Patients with CMC conditions accounted for 59 index visits (42%) and 43 s HCVs (51%). We plotted out the patients’ healthcare trajectories in a department-oriented model, in which PED use (time = T_0_), relative to the previous (time = T_− 1_ ***∈*** (T_*0*_–7 days;T_0_)), second (time = T_1_ ***∈*** (T_0_;T_0_+ 30 days)) and subsequent uses of healthcare (time = T_2_ ***∈*** (T_1_;T_1_+ 30 days)), then time = T_X_ ***∈*** (T_(X-1)_;T_(X-1)_ + 30 days)) were represented graphically (Fig. 3).

The median time between the index visit and unplanned second HCVs was four days (Q1 = 2; Q3 = 16) and the chief complaints described were linked to the index visit in 57% of cases. Four patients attended subsequent visits after these second visits. The patients concerned attended one to six subsequent visits each, some of which were planned and some of which were unplanned. A multivariate analysis including CMC status (independent variable), adjusted for age (independent variable), gave an incidence rate ratio (IRR) for second HCVs (dependent variable) of 1.51 [95% CI: 0.98–2.32], an IRR for planned second HCVs of 1.20 [95%CI 0.76–1.90] and an IRR for unplanned second HCVs of 2.81 [95% CI: 1.20–6.58].

### Healthcare consumption

The median duration of stay for the index visits to the PED was 134 min (Q1: 66 min; Q3: 216 min); 67 index visits concerned patients with osteogenesis imperfecta and, in 20% of cases, the patient was not identified as having a RBD in the medical records of the PED.

The chief complaints recorded were heterogeneous and were linked to the chronic nature of the RBD in 60% of cases. Most of the complaints were orthopedic (53%, *n* = 75), particularly for children with osteogenesis imperfecta (*n* = 50). They included traumatic (45%, *n* = 64) and non-traumatic complaints (8%, *n* = 11), such as limb pain and limping. Infectious complaints, including fever (18%, *n* = 25) were the second most frequent type of complaint. We also assessed two visits for wandering diagnoses resulting in the diagnosis of a RBD at the PED and its confirmation by the reference center. Additional healthcare consumption during index visits occurred in 77% (*n* = 108) of cases and mostly involved interventions by the medical subspecialist (57%; *n* = 80 visits), mostly orthopedic consultations (38%, *n* = 54), and medical imaging (45%, *n* = 64) (Table 2). Twenty-five of the 64 visits for traumatic causes led to orthopedic treatment. The instructions delivered by the PED physician on discharge related to symptomatic (31%) or orthopedic (16%) treatment and clinical monitoring (16%). In 57% (*n* = 80) of index visits, the patients were told to monitor their condition, but were not prescribed any treatment.

**Table 2:** Main characteristics of visits to the pediatric emergency department (PED)

Characteristics	All index visits (*n* = 141)	Index visits without second HCVs (*n* = 57)	Index visits with second HCVs (*n* = 84)	*p*-value	Incidence [95% CI]	HCV incidence rate ratio with CMC status adjusted for age [95% CI]
Median duration of stay in PED (min) [Q1-Q3]	134 [66–216]	116 [66–212]	151 [68–231]	–	–	–
HCVs 7 days before index visit to the PED	44 (31%)	18 (32%)	26 (31%)	0.94	–	–
Patient referred by a healthcare professional	26 (18%)	–	–	–	–	–
Osteogenesis imperfecta	67 (48%)	26 (46%)	41 (49%)	0.71	–	–
Patient not identified as having a RBD	28 (20%)	15 (26%)	13 (15%)	0.11	–	–
Chief complaint linked to chronic condition	85 (60%)	29 (51%)	56 (67%)	0.11	–	–
Chronic medically complex disease	59 (42%)	15 (26%)	43 (51%)	< 0.01	–	–
Healthcare consumption at the PED	108 (77%)	39 (68%)	69 (82%)	0.06	–	–
Subspecialist intervention during PED visit	80 (57%)	27 (47%)	53 (63%)	0.06	–	–
Visit to the PED without treatment	80 (57%)	37 (65%)	43 (51%)	0.11	–	–
Discharged after visit	125 (89%)	50 (88%)	75 (89%)	0.77	–	–
Hospitalization in SSU	8 (6%)	4 (7%)	4 (5%)	0.57	–	–
Hospitalization in conventional ward	10 (7%)	7 (4%)	6 (7%)	0.30	–	–
Second HCVs	84 (60%)	–	–	–	0.60 [0.48–0.74]	1.51 [0.98–2.32]
Planned second HCVs	–	–	60 (71%)	–	0.43 [0.33–0.55]	1.20 [0.76–1.90]
Unplanned second HCVs	–	–	24 (29%)	–	0.17 [0.11–0.25]	2.81 [1.20–6.58]

## Discussion

Repetitive healthcare consumption has an impact on the quality of life of patients and their families (financial cost, absences from school, psychosocial distress), particularly for the patients with the severe and complex conditions [3, 6]. We found that PED visits for children with RBD were associated with other healthcare consumption. These index visits were preceded by a previous HCV in a third of cases and the incidence of a second HCV was 0.60. An analysis of unplanned second HCVs, taking into account CMC status adjusted for age, revealed a significant association between CMC status and the incidence of unplanned second HCVs, with an IRR of 2.81 [95% CI: 1.20–6.58], highlighting the burden of visits to the PED for the most severely affected patients. RDBs are complex diseases with multivisceral involvement. Unsurprisingly, patients with CMC disease accounted for a large proportion (38%) of our study population. Bucholz et al. assessed trends in general pediatric hospitalizations and readmissions at national level in the USA from 2010 to 2016 and showed that the total number of pediatric admissions is declining, but that the complexity of the conditions of the pediatric patients managed by hospitals is increasing. They reported an increase in the 30-day readmission rate over time, from 6.26% in 2010 to 7.02% in 2016, associated with an increase in the numbers of patients with medical complexity, who are at higher risk of readmission. There are probably multiple reasons for this. Children with chronic conditions may be increasing in number due to improvements in survival and advances in medical care, leading to additional medical needs, but this previous study was limited by the exclusion of observation stays and PED visits [22].

No published study has focused on the healthcare trajectories of children with rare diseases visiting the PED. Our results show that the role of the PED in the care of RBD patients is largely one of diagnosis, with much of the healthcare provided in the PED consisting of complementary examinations (77%) and the intervention of medical subspecialists (57%). The PED plays a much lesser role in treatment, with 57% of patients discharged without a prescribed treatment. Nevertheless, the chief complaint at second unplanned HCVs was linked to the index visit in 57% of the cases. Given the high incidence of unplanned second HCVs (IR = 0.17), treatment at the index visit may be inappropriate or insufficient or misdiagnosis may occur, despite the resources deployed during the index visit. We did not assess the preventability of second HCVs. According to Toomey et al., almost 30% of readmissions to a children’s hospital within 30 days may be preventable. However, one of the limitations of secondary HCVs as a means of measuring healthcare quality is that many secondary HCVs are not causally related to the hospital care provided during the index visit. Instead, they may reflect a worsening of underlying disease. In addition, some outpatient (e.g., nurse unable to visit the patient’s home) or patient (e.g., not taking prescribed drugs) factors may contribute to secondary HCVs [23].

Our results indicate that, despite the pathways set up by physicians specializing in RDB and despite the widespread use of subspecialist interventions during PED visits, 17% of index visits are followed by an unplanned second HCV within 30 days. This finding highlights the room for improvement in the healthcare provided to patients with RBD at the PED. There is currently no formalized pathway for families to seek assistance from the physicians of the reference center. Families can send an e-mail or phone text message, but this approach, although effective, remains a bit haphazard because there is no centralization and quantification of this activity. We suggest that RBD reference center physicians could contact the patient systematically 10 days after their visit to the PED, by telephone or telemedicine approaches, and that such contact might limit the number of unplanned HCVs. However, Auger et al. evaluated the effects of a pediatric transition intervention (a single home visit by the nurse) after acute-care hospitalization on post-discharge outcomes in a randomized controlled trial. Contrary to their hypothesis, they found that children assigned to the intervention group had higher rates of unplanned HCVs in the 30 days following discharge. The authors stressed that secondary healthcare consumption is a complex metric resulting from the intersection of many factors [24].

We show here that temporal data are essential for the analysis of healthcare trajectories. New clinical data warehouse tools facilitated the cross-referencing of data, the tracing of healthcare trajectories and the construction of a graphic representation based on an analysis of patient flow. We developed a model of department-oriented healthcare trajectories (Fig. 3). This model reveals possibilities for improvement, by analyzing the flow of passages and the rates of unplanned visits following a visit to the PED. Ultimately, we aim to develop an automated tool for temporal analysis and graphic representation centered on a healthcare act and displaying upstream and downstream healthcare acts. This tool would make it possible to study the healthcare trajectories of patients with rare diseases in a department-oriented model.

Our results also revealed room for improvement in the recognition of children at higher risk in the PED. Indeed, in 20% of index visits, the patients were not identified as having a RBD in the medical records of the PED. The main reason for this was that the family was unaware of the diagnosis and merely indicated that the child was followed in the genetics department. However, the emergency department physician may also not have questioned the family sufficiently precisely about the patient’s medical history. Our patient selection methodology made it possible to identify these patients as having a RBD. This suggests that an automatic tool for collecting the patient’s medical history within emergency patient management software might be of benefit to such patients.

## Limitations

The study was a single-center study and may therefore be subject to selection bias. The study population reflected the diversity of RBD, with a major subgroup corresponding to patients with osteogenesis imperfecta (OI) (39%), which accounted for 48% of index visits (*n* = 67), but was not representative of other RBDs in terms of the chief complaints reported. Indeed, most of the visits involving OI patients concerned orthopedic complaints (*n* = 50).

This study was based on a review of medical records. The quality of the data is therefore dependent on the physician recording the data, potentially generating a measurement bias. The data for unplanned visits after a PED visit may also be incomplete if the patients attended consultations elsewhere. The minimization of this follow-up bias would require an exhaustive study and access to the National Health Insurance database, to trace all healthcare activity for each patient.

The CMC classification of Simons et al. was applied by two members of the research team, and we did not evaluate inter- and intrarater variability for this classification.

Physicians from the reference center for RBDs are contacted daily by families and some aspects are managed by telephone, e-mail, or semi-emergency programmed visits. Patients also have a therapeutic education plan and the e-health mobile phone application RADIOSCAR© (https://www.filiere-oscar.fr/appli-mobile-radioscar) developed by the national RBD network, which allows them to share medical images with RBD physicians. This activity is not quantified. Parents may also take care of their child at home while waiting for a specialist orthopedics consultation the following day, without attending the PED.

## Conclusion

Consistent with our hypothesis, visits to the PED by RBD patients were strongly associated with other healthcare consumption after the index visit. We highlight the patients’ healthcare trajectories in a department-oriented model with a graphical representation of the PED. The IRR of unplanned second HCVs revealed a significant association between CMC status and the incidence of unplanned second HCVs, highlighting the burden of HCVs for the patients with the most chronic and complex disease. However, the results for second and subsequent HCVs reflect the conjunction of many factors. Further prospective studies are required to assess the risk factors for second and subsequent HCVs and to validate the proposed graphical representation of the healthcare trajectory of these patients.

## Supplementary information

**Additional file 1.** Classification of 3 levels of medical complexity. Additional file containing the definition of medical complexity according to Simon et al., 2014 [20].

**Additional file 2.** List of rare bone diseases diagnoses. This table contains the list and frequency of rare bone disease diagnoses in the children included.

## Data Availability

The datasets generated and analysed during the current study are not publicly available due to data privacy reasons but are available from the corresponding author upon reasonable request.

## Abbreviations

CEMARA: *Centre des Maladies Rares*
CI: Confidence interval
CMC: Chronic medically complex
DrWH: Dr. WareHouse®
HCVs: Healthcare visits
IR: Incidence rate
IRR: Incidence rate ratio
NEM: Necker Enfants-Malades Hospital
PED: Pediatric Emergency department
RBD: Rare bone disease
