# Supplementary material for: Healthcare trajectory of children with rare bone disease attending pediatric emergency departments
Source: Orphanet J Rare Dis. 2020 Jan 3;15:2. doi: 10.1186/s13023-019-1284-1 (PMC6942261; doi:10.1186/s13023-019-1284-1)
Supplement: Supplementary file 1 — Additional file 1. Classification of 3 levels of medical complexity. Additional file containing the definition of medical complexity according to Simon et al., 2014 [20]. [file 13023_2019_1284_MOESM1_ESM.docx]

**Additional file 1**

**Classification of 3 levels of medical complexity (Simon et al, 2014)**

**Children with Complex Chronic Disease** :
Significant chronic conditions in 2 body systems. Significant chronic condition is defined as a physical, mental or developmental condition that can be expected to last at least a year, will use health care resources above the level for a healthy child, require treatment of control of the condition, and the condition can be expected to be episodically or continuously debilitating.

Body systems include: cardiac, craniofacial, dermatologic, endocrinologic, gastrointestinal, genetic, genitourinary, hematologic, immunologic, mental health, metabolic, musculoskeletal, neurologic, ophthalmologic, otologic, pulmonary/respiratory, and renal.

OR A progressive condition that is associated with deteriorating health with a decreased life expectancy in adulthood.

OR Continuous dependence on technology for at least 6 months.

OR Malignancies: progressive or metastatic malignancies that affect life function. Exclude those in remission for .5 years.

**Children with Noncomplex Chronic Disease** :
Chronic conditions that last at least 1 year: These conditions are commonly lifelong but can be episodic with periods of good health between episodes. They include physical, developmental, or mental health conditions that may persist into adulthood but may also resolve either secondary to the natural history of the disease or as a result of surgical intervention. These conditions involve a single body system, are not progressive, can vary widely in severity, and result in highly variable health care utilization.

**Children without Chronic Disease** :
Acute nonchronic conditions: A physical, developmental or mental health condition that is not expected to last .1 year. These children may temporarily (for ,1 year) use health care resources above the normal level for a healthy child.

OR Healthy: No acute or chronic health conditions. These children do not use health care resources above the normal level for a healthy child.
