# Supplementary material for: Healthcare trajectory of children with rare bone disease attending pediatric emergency departments
Source: Orphanet J Rare Dis. 2020 Jan 3;15:2. doi: 10.1186/s13023-019-1284-1 (PMC6942261; doi:10.1186/s13023-019-1284-1)
Supplement: Supplementary file 2 — Additional file 2. List of rare bone diseases diagnoses. This table contains the list and frequency of rare bone disease diagnoses in the children included. [file 13023_2019_1284_MOESM2_ESM.docx]

## **Additional file 2:** List of rare bone diseases diagnosis

| Rare bone disease | Number of visit in Pediatric emergecy room | (%) | Number of patient in study population | (%) |
| --- | --- | --- | --- | --- |
| Osteogenesis imperfecta | 81 | 46% | 37 | 39% |
| Fanconi anemia | 10 | 6% | 1 | 1% |
| Goldenhar syndrome | 9 | 5% | 3 | 3% |
| Cleidocranial dysplasia | 5 | 3% | 1 | 1% |
| Pallister-Hall syndrome | 5 | 3% | 1 | 1% |
| Gorham-Stout disease | 4 | 2% | 1 | 1% |
| Multiple osteochondromas | 4 | 2% | 3 | 3% |
| Achondroplasia | 3 | 2% | 3 | 3% |
| Cornelia de Lange syndrome | 3 | 2% | 1 | 1% |
| Craniosynostosis | 3 | 2% | 3 | 3% |
| Genitopatellar syndrome | 3 | 2% | 1 | 1% |
| Holt-Oram syndrome | 3 | 2% | 2 | 2% |
| Metatropic dysplasias | 3 | 2% | 1 | 1% |
| Treacher-Collins syndrome | 3 | 2% | 2 | 2% |
| Acrodysostosis | 2 | 1% | 1 | 1% |
| Adams-Oliver syndrome | 2 | 1% | 2 | 2% |
| Mucopolysaccharidosis type 4A | 2 | 1% | 1 | 1% |
| Multiple metaphyseal dysplasia | 2 | 1% | 2 | 2% |
| Primary bone dysplasia with multiple joint dislocations | 2 | 1% | 1 | 1% |
| Pycnodysostosis | 2 | 1% | 1 | 1% |
| Acrocephalosyndactyly | 1 | 1% | 1 | 1% |
| Caffey disease | 1 | 1% | 1 | 1% |
| Crouzon disease | 1 | 1% | 1 | 1% |
| Dyssegmental dysplasia, Silverman-Handmaker type | 1 | 1% | 1 | 1% |
| Enchondromatosis | 1 | 1% | 1 | 1% |
| Fibrodysplasia ossificans progressiva | 1 | 1% | 1 | 1% |
| Greig cephalopolysyndactyly syndrome | 1 | 1% | 1 | 1% |
| Hurler syndrome | 1 | 1% | 1 | 1% |
| Hypochondroplasia | 1 | 1% | 1 | 1% |
| Isolated Klippel-Feil syndrome | 1 | 1% | 1 | 1% |
| McCune-Albright syndrome | 1 | 1% | 1 | 1% |
| Metaphyseal chondrodysplasia, Schmid type | 1 | 1% | 1 | 1% |
| Mucolipidosis type II | 1 | 1% | 1 | 1% |
| Multiple epiphyseal dysplasia | 1 | 1% | 1 | 1% |
| Nail-patella syndrome | 1 | 1% | 1 | 1% |
| Oculoauriculovertebral spectrum with radial defects | 1 | 1% | 1 | 1% |
| Pfeiffer syndrome | 1 | 1% | 1 | 1% |
| Poland syndrome | 1 | 1% | 1 | 1% |
| Rubinstein-Taybi syndrome | 1 | 1% | 1 | 1% |
| Saethre-Chotzen syndrome | 1 | 1% | 1 | 1% |
| Schimke immu0-osseous dysplasia | 1 | 1% | 1 | 1% |
| SPONASTRIME dysplasia | 1 | 1% | 1 | 1% |
| Spondylometaphyseal dysplasia | 1 | 1% | 1 | 1% |
| Stickler syndrome | 1 | 1% | 1 | 1% |
| Unclassified metaphyseal chondrodysplasia | 2 | 1% | 2 | 2% |
|  |  |  |  |  |
| Total | **177** |  | **94** |  |
